# Lung Cancer Assistant: a hybrid clinical decision support application for lung cancer care

**DOI:** 10.1098/rsif.2014.0534

**Published:** 2014-09-06

**Authors:** M. Berkan Sesen, Michael D. Peake, Rene Banares-Alcantara, Donald Tse, Timor Kadir, Roz Stanley, Fergus Gleeson, Michael Brady

**Affiliations:** 1 Department of Engineering Science, University of Oxford, Oxford OX1 3PJ, UK; 2 Clinical Effectiveness and Evaluation Unit, Royal College of Physicians of London, London NW1 4LE, UK; 3 Department of Respiratory Medicine, Glenfield Hospital, Leicester LE3 9QP, UK; 4 Department of Clinical Radiology, Oxford University Hospitals NHS Trust, Oxford OX3 7LJ, UK; 5 Mirada Medical, Oxford OX1 1BY, UK; 6 Department of Oncology, University of Oxford, Oxford OX3 7DQ, UK

**Keywords:** medical informatics, computerized decision support, lung cancer care

## Abstract

Multidisciplinary team (MDT) meetings are becoming the model of care for cancer patients worldwide. While MDTs have improved the quality of cancer care, the meetings impose substantial time pressure on the members, who generally attend several such MDTs. We describe Lung Cancer Assistant (LCA), a clinical decision support (CDS) prototype designed to assist the experts in the treatment selection decisions in the lung cancer MDTs. A novel feature of LCA is its ability to provide rule-based and probabilistic decision support within a single platform. The guideline-based CDS is based on clinical guideline rules, while the probabilistic CDS is based on a Bayesian network trained on the English Lung Cancer Audit Database (LUCADA). We assess rule-based and probabilistic recommendations based on their concordances with the treatments recorded in LUCADA. Our results reveal that the guideline rule-based recommendations perform well in simulating the recorded treatments with exact and partial concordance rates of 0.57 and 0.79, respectively. On the other hand, the exact and partial concordance rates achieved with probabilistic results are relatively poorer with 0.27 and 0.76. However, probabilistic decision support fulfils a complementary role in providing accurate survival estimations. Compared to recorded treatments, both CDS approaches promote higher resection rates and multimodality treatments.

## 1. Introduction

Multidisciplinary teams (MDTs) are becoming the model of care for cancer patients worldwide [1]. The immediate benefit of these meetings is their ability to facilitate collective thinking and expertise sharing, as opposed to the outdated sequential management by a series of clinicians in isolation [2]. There are around 1500 cancer MDTs in the UK, meeting weekly in different centres across the country [3]. Similar to other cancers, lung cancer MDTs generally consist of oncologists, histopathologists, radiologists, specialist nurses and thoracic surgeons along with consultant respiratory physicians. The decisions are usually made based on published research evidence, relevant clinical guideline recommendations, and the shared expertise of the team members from previous similar cases.

An increasingly significant observation is that the volume of data that needs to be processed in an MDT meeting is not only large and variable, but also comes from different sources, making consolidation more difficult. Adding to the complexity of the situation, most MDTs work against tight time schedules, and often need to determine the best treatment option within a matter of minutes. Consequently, the MDT is inherently prone to errors, primarily because relevant information may not be considered. In 2011, Lamb *et al.* reported that excessive workload and time pressure were the two most detrimental factors that lower team morale, reduce attendance, and rush decision-making [1]. In a similar vein, Lanceley *et al*. argued that MDT meetings suffer from unstructured case discussion, time pressure and variability in the quality of decision-making [4].

In order to reinforce the diligence and expertise of the clinicians, clinical decision support (CDS) systems have been developed. These are computer-based tools that provide assistance in synthesizing and integrating patient-specific information and presenting recommendations to clinicians at the point of care [5]. As objective decision aides that can match patient data to medical knowledge, their purpose is to assist, rather than to replace the clinician. Winning the cooperation of the clinicians is crucial for wider adoption of CDS. Previous research shows that clinicians accept computer warnings and recommendations but resist processes that interfere with their daily workflow or challenge their autonomy [6].

CDS systems have been implemented in many different clinical settings, where the decision-making process is error-prone due to the diversity of medical information and the uncertainties associated with it [7]. In addition to the uncertainties, the excessive workload and time constraints of the team members mean that the MDT meeting exemplifies a clinical context that is ideal for CDS implementation. A CDS system that can consolidate information from different sources, while also dealing with uncertainty in a precise and mathematically sound way, can be employed to provide patient-specific and evidence-based recommendations in order to reduce the time pressures of the team, better structure the patient case discussions and ensure that errors of omission are minimized.

Current practice in CDS for MDTs is guideline rule-based systems that help reduce the gap between clinical evidence and practice by facilitating the adoption of clinical guideline rules within the MDT meetings. To date, MDTSuite [2] in colorectal cancer and MATE [8] in breast cancer have been the major guideline rule-based CDS applications that have been researched and applied in clinical pilot studies. In delivering evidence-based decision support, such systems use different computer interpretable guideline (CIG) formalisms that operate on the principle of matching individual patient entries to a set of computerized clinical guideline rules in order to generate patient-specific arguments that support or oppose particular treatment options.

This argumentation-based decision model has the benefit of laying out all treatment options clearly and making evidence explicit. However, a strictly guideline rule-based approach to CDS also has certain limitations. First, such systems are imprecise in quantifying the statistical or probabilistic level of support associated with different treatment options. Second, the elicitation and maintenance of the rule-based domain representations of such systems are expensive and time-consuming. In reality, covering the entire disease domain using only clinical guideline rules is highly challenging [2].

An alternative to the deterministic approach for representing and reasoning with domain knowledge employed by such rule-based systems is probabilistic inference. Unlike rule-based systems, probabilistic models trained on existing patient data are able to provide quantified and more precise answers to survival-related queries. While their inference mechanisms are usually less explicit than argumentation-based decision models, there are exceptions, not least Bayesian networks (BNs), which enable probabilistic inference in a visually more appealing and transparent way. A BN consists of two components: a directed acyclic graph (DAG) that defines the probabilistic dependencies between different nodes and a joint probability distribution that represents the entire probability space of the domain. To date, [9] in colon cancer, [10] in skeletal metastases and [11] in lung cancer form the largest studies in survival prediction in cancer care using BNs. Unfortunately, such probabilistic applications are not widely available since they rely on the availability of electronic patient data, which are still a rarity.

Motivated by the increasing clinical need for CDS in MDT meetings and the limitations of the conventional rule-based CDS applications, in this paper we introduce an online CDS application, *Lung Cancer Assistant* (*LCA*), which combines rule-based and probabilistic inference in order to aid clinicians to arrive at more informed treatment selection decisions in the lung cancer MDT meetings. The online platform is accessible through the LCA website (http://www.lca.eng.ox.ac.uk).

## 2. Material and methods

We first introduce the English National Lung Cancer Audit Database (LUCADA), on which we base our system design and empirical results. We then present the methodologies through which we achieved semantic and probabilistic inference within LCA.

### 2.1. LUCADA database

Since 2004, the National Lung Cancer Audit (NLCA) has been collecting data on lung cancer patients diagnosed in England. The data are collected via a secure web portal with password restricted access, using a clinically designed dataset and stored in a central database known as LUCADA. It is aimed at providing a better understanding of the care delivered during referral, diagnosis and treatment of lung cancer patients and how that impacts on patient outcomes, particularly survival [12,13]. Individual hospital trusts can either enter data directly or upload using CSV or XML files.

Through a data-sharing agreement between the NLCA team and the University of Oxford, we have had access to an anonymized subset of the LUCADA, including 126 986 English patient records entered into the system from the beginning of 2006 until the end of 2010. This approximates to 95% of all English patients entered into the system for the given time period.

In this study, we focus on patients with lung cancer including those with diagnoses of non-small cell lung cancer (NSCLC) and small cell lung cancer confirmed by a tissue diagnosis plus those patients diagnosed only on clinical grounds. We exclude mesothelioma patients from the study since it is a different disease for which we had fewer patient records and UK published guidelines.

A complete list of all LUCADA variables, along with their full definitions and the list of values they can take, is given in the LUCADA Data Manual document [14] available on the web. Table 1 recalls the 13 patient- and disease-specific variables that we chose to include in our studies from the LUCADA dataset. These can be grouped into three categories with respect to their temporal order in the patient journey, namely: ‘pre-treatment (1–11)’, ‘treatment (12)’ and ‘outcome (13)’ variables.

**Table 1.** The 13 patient- and disease-specific variables from LUCADA, along with the values they can take and their temporal orders.

code	name	values
1	age	<50; 50–60; 60–70; 70–80; >80
2	staging identifier	6; 7
3	FEV1^a^ absolute amount	<1.0; 1–1.5; 1.5–2.0; >2.0
4	FEV1 percentage	<30; 30–40; 40–80; >80
5	performance status	0; 1; 2; 3; 4
6	number of comorbidities	0; 1; 2; 3; 4; 5
7	primary diagnosis	C33; C34; C34.0; C34.1; C34.2; C34.3; C34.8; C34.9; C38.4; C38.3; C38.8
8	tumour laterality	left; right; midline; bilateral; n.a.
9	TNM category	IA; IB; IIA; IIB; IIIA; IIIB; IV; Uncertain
10	histology	M8010/2; M8041/3; M8046/3; M8070/3; M8140/3; M8250/3; M8012/3; M8020/3; M8013/3; M8240; M8980/3; M8940/3; M9999/9
11	site-specific staging classification	limited; extensive; unknown
12	suggested cancer treatment plan	listed in table 2
13	1-year survival	alive; dead

^a^Forced expiratory volume in 1 s.

The meanings of most of the terms are self-evident to a clinician, though these are spelled out in more detail in [11], to which the reader is referred to. We note that in table 1, the ‘1-year survival’ variable contains the survival outcome information for all patient records. In cancer care, long-term disease-free survival is more commonly reported with a cut-off point of 5 years. Owing to lack of sufficient 5-year survival data in LUCADA, we use 1-year survival as a surrogate outcome measure. This choice is also supported by the literature, which reports almost all improvement in lung cancer survival as being attributable to an increase in 1-year survival [15,16]. The overall ‘1-year survival’ rate within LUCADA is 33%. Table 2 lists the available treatment options with their frequencies in LUCADA.

**Table 2.** The available treatment plan options and their frequencies in LUCADA.

code	name	percentage (%)
1	surgery	10
2	radiotherapy	14.79
3	chemotherapy	19
5	palliative care	23
6	active monitoring	9
7	sequential chemotherapy and radiotherapy	7
8	concurrent chemotherapy and radiotherapy	1
9	induction chemotherapy to downstage before surgery	0.08
10	neo-adjuvant chemotherapy and surgery	0.13
11	surgery followed by adjuvant chemotherapy	2
—	null	14

### 2.2. Guideline rule-based decision support

In order to reap the benefits of clinical guidelines, they need to be easily accessible at the point of care by clinicians [17]. CIGs enable computerizing guideline eligibility and decision criteria in order to deliver patient-specific recommendations to clinicians [18]. Among the relevant MDT CDS projects listed, MATE used a CIG formalism named PROforma [8] with a proprietary execution engine, with no active support at the time of our research. In addition, PROforma did not allow integration with an external ontology. On the other hand, MDTSuite used a more recent approach based on resource description framework (RDF) triplets and queries. While this RDF query-based framework was able to infer subsumption relationships such as ‘Lung Cancer is a Cancer’, it did not allow reasoning with description logic (DL) axioms. For detailed comparisons of the commonly used CIG formalisms, the reader is referred to [19–21].

**Figure 1.**
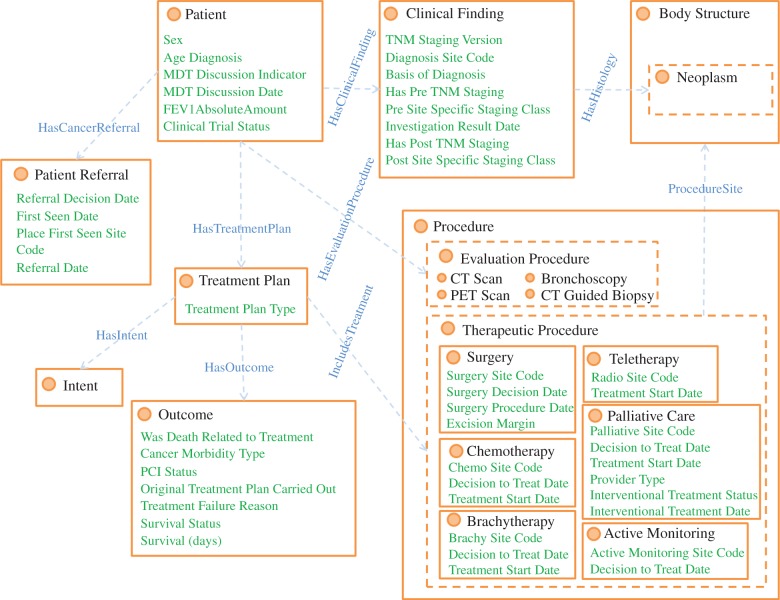
The LUCADA ontology clinical domain T-box. The circles represent clinical classes; the edges represent object properties between these classes and the list items represent the datatype properties belonging to the respective classes. (Online version in colour.)

Within LCA, we captured the domain knowledge by using a domain-specific clinical ontology. In general, ontologies are well suited to classify and encode semantic relationships between domain concepts. To this end, we designed a local ontology that conceptualizes the LUCADA data model [14], using the Web Ontology Language 2 (OWL 2). The terminological box (T-box) of this ontology is given in figure 1. OWL-2 is the ontology language officially endorsed by the World Wide Web Consortium (W3C). It is based on DL, allowing the use of semantic inferences through the use of axioms.

Subsequently, we performed mappings between the local ontology classes and SNOMED-CT [22] concepts with the help of Logmap-2 [23]. SNOMED-CT is the *lingua franca* of medicine [24] and also the full fundamental standard for all medical information applications within the NHS. Following the mapping, we used the Locality Module Extractor tool [25] for extracting a minimal and complete module of SNOMED-CT, referred to as the LUCADA ontology for the remainder of the text. This module preserves all semantic information relevant to our mapped concepts.

However, some classes did not have one-to-one mappings with a SNOMED-CT concept. Some of these were modelled as post-coordinated concepts making use of the atomic concepts and attributes available in SNOMED-CT as explained in [26]. Overall, out of the 376 concepts that we extracted from the LUCADA data model, 13 could not be mapped to SNOMED-CT and were kept as proprietary classes in the LUCADA ontology.

**Figure 2.**
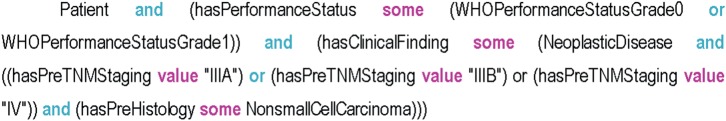
The class equivalence axiom describing the eligibility criteria for patients with Stage I, II, III disease and good performance status. (Online version in colour.)

In addition to our ontological domain representation, we devised an ontological guideline rule inference framework in order to draw inferences from guideline rules. According to this, we represent the guideline rule antecedents as defined patient scenario classes, whose equivalent class descriptions capture the semantics for rule eligibility criteria. As an example, the eligibility for the guideline rule (taken from the NICE guideline document [27]) ‘Consider radiotherapy for Stage I, II, III patients with good performance status’ is encoded as the OWL 2 class equivalence axiom in figure 2, which makes use of concepts and properties in the LUCADA ontology, along with existential and universal DL constraints [28].

**Figure 3.**
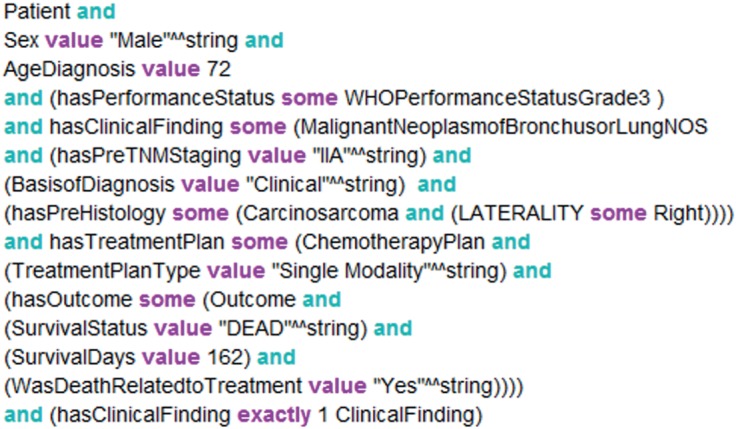
An automatically created OWL DL query for a patient record from the LUCADA database. (Online version in colour.)

We represent a patient record in the form of a DL query that is automatically parsed by LCA using the Java OWL API [29]. A demonstrative representation of a LUCADA patient record as a DL query is provided in figure 3. The DL query acts as a pseudo class definition, and as such ontological inference can be achieved at the T-box level, merely by classifying the ontology. This allows faster inference times compared with ontological inference with individuals at the assertional box level [28].

We make use of the FaCT++ semantic reasoner [30] to infer the patient scenario class memberships of the automatically generated patient-DL query. The parent patient scenario classes, for which the patient satisfies the eligibility criteria, represent the list of guideline rules that apply to the given patient. Similar to the argumentation-based decision model employed by MATE [8] and MDTSuite [2], we produce patient-specific arguments, which are in favour of or against a given treatment option. These are then aggregated within the LCA decision support engine to compare different treatment options and recommend to the user the treatment that has the highest net support.

Among the better known CIG formalisms, EON, GLIF3 and SAGE are the main ones that support a similar use of external ontologies for conceptualization and data abstraction. However, GLIF3 and EON are discontinued and SAGE is now proprietary. Our adoption of OWL as the guideline expression language allowed us to represent clinical knowledge in a standardized, open source format and carry-out inferences using a publicly available and well-maintained semantic reasoner.

It should, however, be mentioned that our framework does not contain temporal concepts to incorporate sequential clinical workflow management as most CIG formalisms do. This is partly due to the LUCADA data model which does not portray the patient journey in a temporal manner, and partly due to the fact that the emphasis of the decision support is for a single meeting and does not involve a series of sequential tasks that would necessitate temporal workflow management. The design of the LUCADA ontology and the ontological guideline rule inference framework are explained in more detail in [26,28].

#### 2.2.1. Guideline rule elicitation

In order to populate the guideline rule base for LCA, we carried out detailed reviews of the four publicly available national and international guideline documents in lung cancer care. As a result of our detailed reviews, we extracted 84 treatment-related rules from (i) the British Thoracic Society (BTS) [31], (ii) National Institute for Clinical Excellence (NICE) [27], (iii) European Society for Medical Oncology (ESMO) [32] and (iv) the National Comprehensive Cancer Network (NCCN) [33] guideline documents.

In general, the narrative language for the guideline rules employed terminology that was open to interpretation, such as ‘operable’ and ‘suitable for concurrent radiotherapy’. We interpreted rules that contained such terminology based on the shared expertise of our clinician collaborators. This formalization of ambiguous terminology is a well-established bottleneck in the development of guideline rule-based CDS applications [2,34].

In addition, we needed to ‘operationalize’ the guideline rules in terms of the LUCADA data model that defined the boundaries of the clinical concepts we could use. In some cases, LUCADA did not encompass all the concepts necessary to entirely capture the semantics of certain rule criteria. For instance, while conceptualizing criteria as ‘resectable’ or ‘operable’ that indicate suitability for resection, we used a patient's performance status and the existence of a cardiovascular co-morbidity as surrogates to the indicators listed in the guidelines, such as risk of peri/post-operative mortality, cardiac functional capacity, lung function and post-operative quality of life [27,31]. These approximations were necessary in order to maximize our use of LUCADA data.

### 2.3. Probabilistic decision support

We soon realized that a purely rule-based decision support approach falls short in answering certain critical questions that the MDT members face on a weekly basis. Confronted with a patient case in the MDT meeting, the answers to the questions of ‘What is the probability of survival for this patient?’ and ‘How would different treatment decisions affect this probability?’ generally drive the decision-making process.

**Figure 4.**
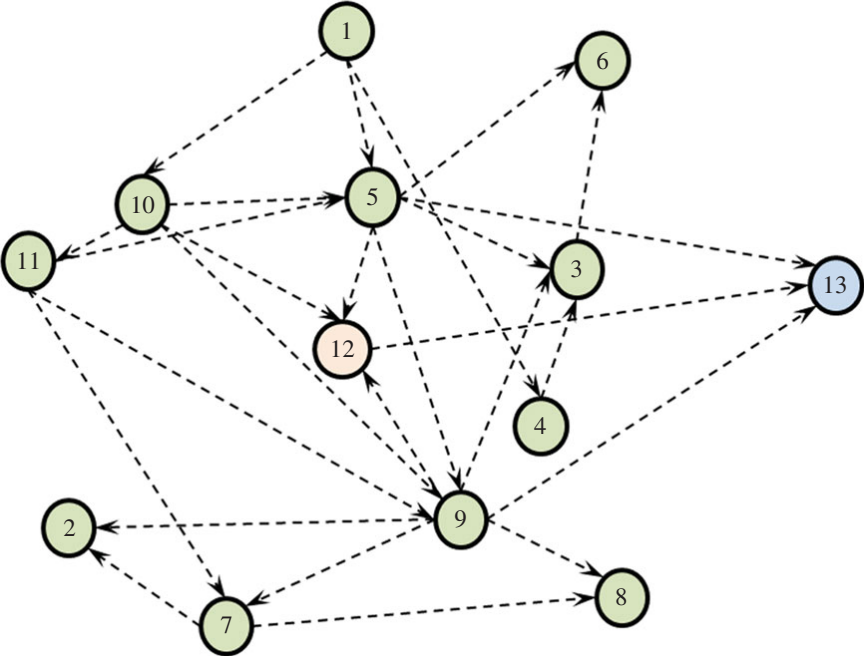
The BN structure used for carrying out causal interventions on the ‘suggested cancer treatment plan’ variable. The nodes follow the same numbering as in table 1. (Online version in colour.)

We have developed a BN in order to provide probabilistic answers to these questions. This required learning the DAG structure that best fits the dataset and represents the domain. In general, structure learning algorithms can be categorized into constraint- and score-based search approaches [35]. In our case, structure learning was performed by incorporating expert elicited and temporal constraints into a score-based approach using stochastic search. This achieved highly accurate survival predictions with an area under the receiver operating characteristic curve (AUC) of 0.81 (±0.03) [11]. The LUCADA BN that we use for the causal interventions is shown in figure 4.

According to the network structure, ‘performance status (5)’, ‘TNM category (9)’ and ‘treatment plan (12)’ are the nodes that are directly related to ‘survival (13)’. The rest of the nodes, however, may provide valuable insight into the joint probability distribution and potentially the causal relations of the domain. By sampling from this distribution, it is also possible to estimate missing data points or generate synthetic ones in the input space, which categorizes BNs as generative models [36]. Missing data are a reality of clinical datasets and the conditional probabilistic dependencies encoded in the DAG allow missing data to be dealt with more efficiently [37]. For detailed information on BNs, along with discussions on the design steps of the LUCADA BN, the reader is referred to [38,39].

### 2.4. System architecture

The architecture of the LCA was informed by our design goals to develop a CDS prototype that can provide instantaneous evidence-based and probabilistic decision support at the point of care, while prioritizing the standardization of domain knowledge and interoperability with other software.

**Figure 5.**
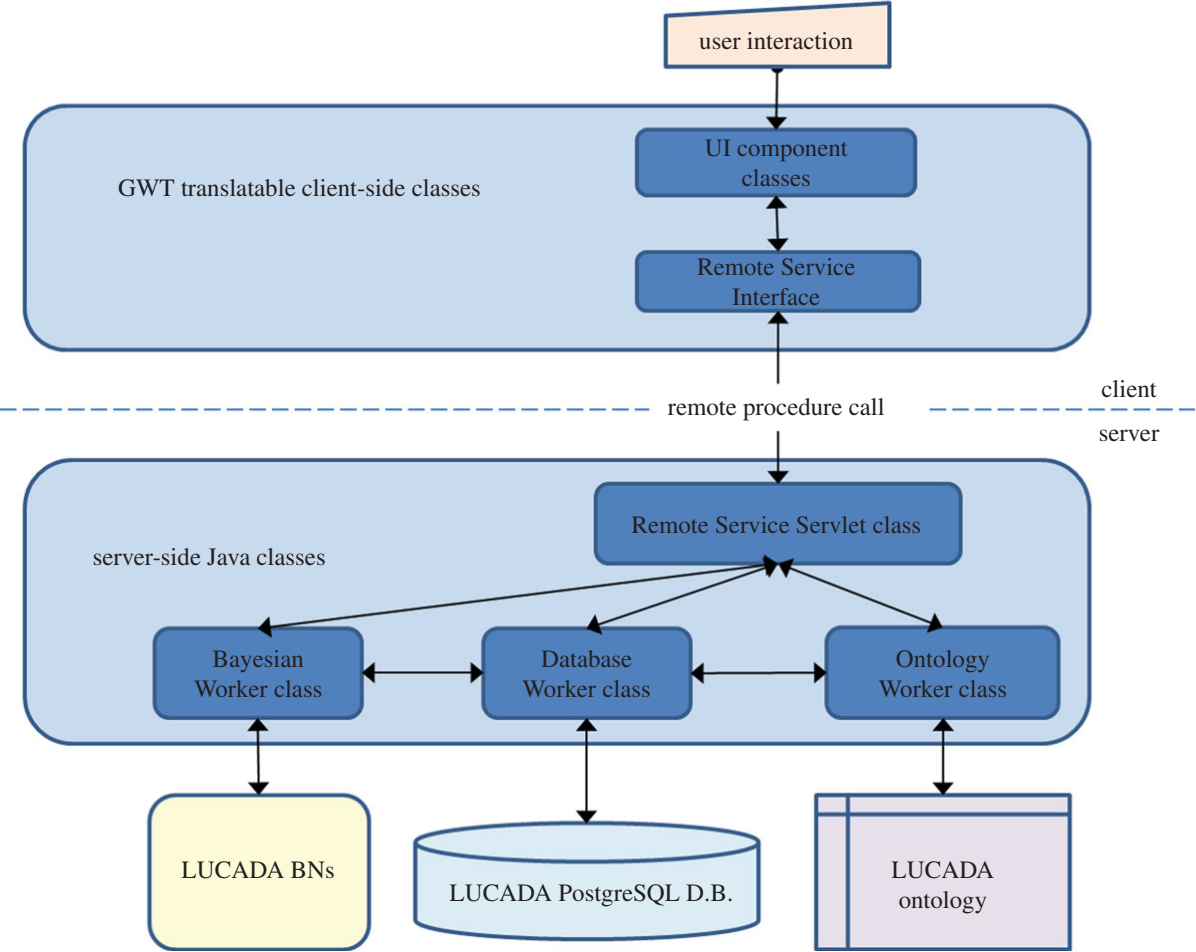
The software architecture of the LCA CDS prototype. (Online version in colour.)

We developed LCA with the Google Web Toolkit software development kit v. 2.4.0 [40] in Java. The software architecture is shown in figure 5. According to this, the user interacts with the CDS prototype through a web-based form. Depending on the nature of a client-side request, the ‘implemented’ Remote Service Servlet class methods make use of the ‘Database Worker’, ‘Ontology Worker’ or ‘Bayesian Worker’ classes in order to perform inference. The Database Worker modifies or queries the database, which is stored in PostgreSQL. It uses JDBC [41] for connecting to and querying or modifying the LUCADA patient records and fulfils a central role, enabling the communication of electronic patient information with both ‘Ontology Worker’ and ‘Bayesian Worker’ classes.

The ‘Ontology Worker’ class mainly uses the OWL API [29] for communicating with and querying the LUCADA ontology with the help of the FaCT++ [30] semantic reasoner. And the ‘Bayesian Worker’ class enables probabilistic reasoning. It allows building and saving BNs in the standardized Bayesian Interchange Format, which is compatible with the majority of commercial and educational BN software tools. More importantly, it contains a bucket tree algorithm [42] implementation that allows probabilistic inference to be performed on the BN in order to return posterior survival probabilities.

## 3. Results

**Figure 6.**
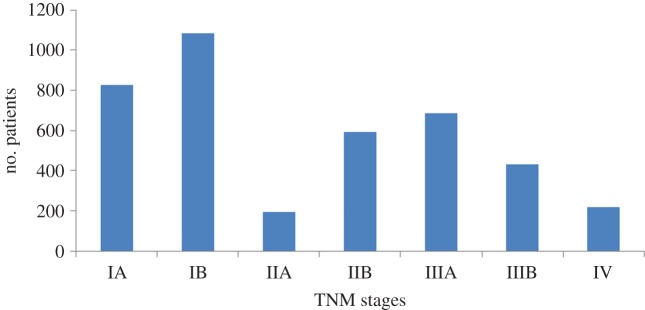
TNM stages distributions of the 4020 patients included in the CDS recommendation experiments. (Online version in colour.)

We ran two sets of experiments to assess the guideline rule-based and probabilistic decision support functionalities of LCA on a carefully selected subset of LUCADA, which only contained patients who were given a curative treatment plan and had no missing data. This resulted in a fully observed subset of 4020 patients. Figure 6 gives a breakdown of these patients with respect to their TNM stages.

**Figure 7.**
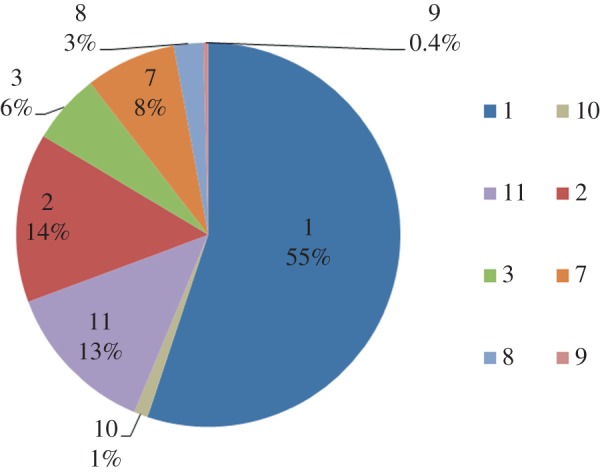
Recorded treatment plan distributions of the 4020 patients included in the CDS experiments. The treatment plan codes are as listed in table 2. (Online version in colour.)

Of the 4020 patient records, which were given curative treatment plans, the recorded treatment plans were distributed as shown in figure 7, which adopts the treatment plan numbering from table 2.

Making use of this patient subset and the two alternative decision support approaches, we evaluated concordance rates with respect to both exact and partial matches between LCA's top treatment recommendation and the treatments recorded in the database. These recorded treatments were adopted as a ‘silver standard’ in the absence of prospective patient data. Partial matches contained patients for whom the top recommendation of the system either subsumed or overlapped with the recorded treatment. For instance, a commonly occurring partial match pattern consisted of patients for which the recorded treatment plan ‘surgery’ was subsumed by the top LCA recommendation ‘surgery followed by adjuvant chemotherapy’.

For guideline rule-based decision support, the top system recommendation was defined as the treatment plan option that had the highest net support. For probabilistic decision support, our major goal was to investigate whether the LCA BN, which produced highly accurate survival estimates, could be used for making plausible treatment recommendations based on maximizing survival.

Overall, the LCA guideline rule-based decision support achieved an exact concordance rate of 0.57 with the recorded treatments in LUCADA, which rose to 0.79 when partial matches were included. On the other hand, the performance of the LCA probabilistic decision support was worse with 0.27 and 0.76 for the exact and partial concordance rates, respectively.

### 3.1. Analyses with respect to treatment plan

**Figure 8.**
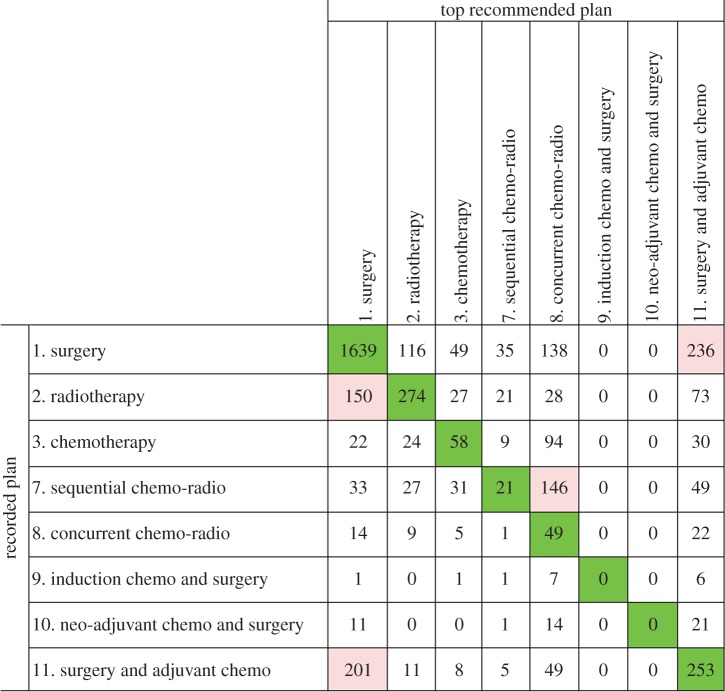
The confusion matrix that displays the recorded treatment plans in the database versus the top guideline rule-based recommendations by LCA. (Online version in colour.)

**Figure 9.**
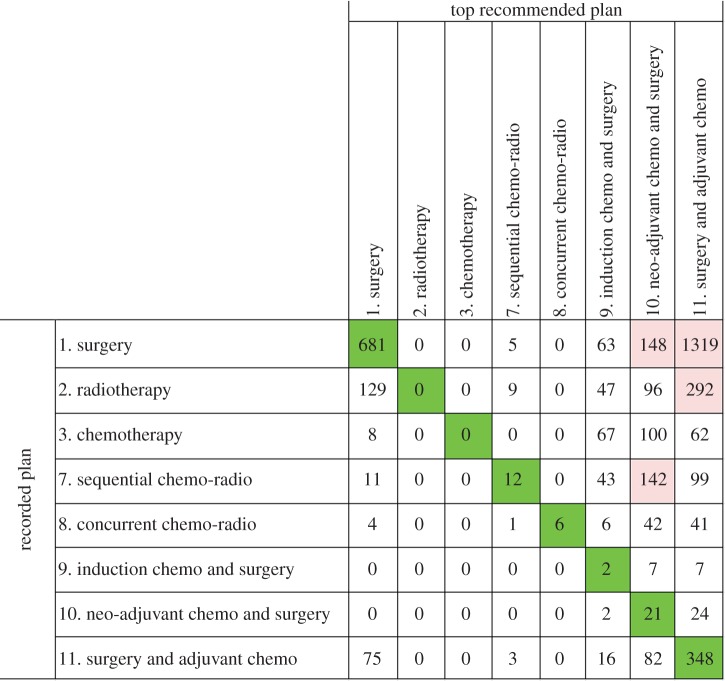
The confusion matrix that displays the recorded treatment plans versus the top probabilistic recommendations by LCA. (Online version in colour.)

Figures 8 and 9 show the confusion matrices that summarize the aggregated discrepancies between the recorded treatment plans and the guideline rule-based and probabilistic decision support, respectively. In both figures, the numbers on the diagonals indicate concordant cases for each treatment plan type.

As an alternative indicator of consistency between the recommendations and the recorded treatments, we provide agreement analyses, using the unweighted kappa statistic [43] and assuming treatment to be a nominal variable with eight possible values for each set of CDS results. Furthermore, we present the proportions of specific agreement [44] and kappa statistics per individual treatment by collapsing the confusion matrices relative to each specific treatment.

For guideline rule-based recommendations, the overall inter-rater agreement with recorded treatments was found to be *κ* = 0.36 (*p* < 0.05, 95% CI (0.34, 0.38)). The proportions of specific agreements and kappa statistics per treatment are given in table 3.

**Table 3.** The kappa statistics and proportions of specific agreements for guideline recommendations per treatment plan type, along with their 95% asymptotic confidence intervals.

	kappa statistic	specific agreement
1. surgery	0.50, (0.47–0.52)	0.77, (0.76–0.78)
2. radiotherapy	0.46, (0.42–0.50)	0.53, (0.49–0.56)
3. chemotherapy	0.24, (0.18–0.30)	0.29, (0.23–0.34)
7. sequential chemo-radio	0.07, (0.03–0.11)	0.10, (0.06–0.14)
8. concurrent chemo-radio	0.12, (0.08–0.16)	0.16, (0.12–020)
9. induction chemo and surgery	0	0
10. neo-adjuvant chemo and surgery	0	0
11. surgery and adjuvant chemo	0.31, (0.28–0.35)	0.42, (0.38–0.46)

Comparing the guideline rule-based recommendations of LCA with the recorded ‘surgery’ treatment plans on row 1 in figure 8, it is evident that the two are concordant for the majority of the cases. The discordances mainly arise due to LCA recommending adjuvant chemotherapy after surgery, whereas the recorded treatment is surgery alone. Upon further analysis, we found that this group consisted entirely of locally advanced stage (Stage IIIA and IIIB) patients, for whom all guideline documents recommend adjuvant chemotherapy after surgery.

On the other hand, if we focus on the ‘adjuvant chemotherapy after surgery’ row, we see that the majority of discordances (201 patients) stem from the system suggesting surgery alone. These patients are entirely early stage patients, and the disagreement of the system stems from a guideline rule stating ‘There is no evidence of benefit of postoperative chemotherapy in stage IA non-small cell lung cancer in a western population’ taken from the BTS document [31]. Again, though debatable, the system's suggestion is defensible.

Another notable pattern in figure 8 is that the discordant portion of the ‘radiotherapy’ column is mainly comprised patients for whom the top guideline-based recommendation was surgery. These may potentially represent complex cases for which suitability for surgery cannot be determined by the guideline rules and information stored in the LUCADA database. Similar low exact concordance percentages are also observable for patients who have been treated with ‘chemotherapy’ and ‘sequential chemo-radiotherapy’.

For the ‘chemotherapy’ group, guideline-based decision support highly favours multimodality treatments, such as ‘concurrent chemo-radiotherapy’ or ‘adjuvant chemotherapy after surgery’, over chemotherapy alone. For patients who have been given ‘sequential chemo-radiotherapy’, the LCA rule base again mainly favours ‘concurrent chemo-radiotherapy’. In the majority of the 146 cases, the discordance is due to a BTS [31] guideline stating that ‘concurrent chemo-radiotherapy is more efficacious and should be preferred to sequential chemo-radiotherapy if the patient is fit enough’. Several NSCLC trials have compared sequential and concurrent chemo-radiotherapy, with almost all reporting increased survival with the latter [45]. However, evidently LCA cannot distinguish between the two, which may be due to the lack of clearly defined criteria of ‘fit enough’. This may indeed be regarded as a typical example of the effects of ambiguous wording in guideline rules that are prone to variable interpretations and may result in the recommendation of a sub-optimal treatment by the system. Therefore, any clinical use of the system would need the guidance and additional advice of an expert oncologist.

Finally, in figure 8, two exceptional patient groups, for whom concordance levels are zero, are those who have been given ‘induction chemotherapy before surgery’ or ‘neo-adjuvant chemotherapy before surgery’. Such discordances derive from the fact that no guideline rules that recommend these two treatment plans currently exist since they are only prescribed under clinical trials. This is a common occurrence in clinical practice, where day-to-day clinical practice often lags behind state-of-the-art treatments until sufficient evidence accumulates.

In contrast with the fairly accurate guideline rule-based recommendations, figure 9 reflects notably poorer concordance results for probabilistic treatment recommendations with an inter-rater agreement of *κ* = 0.09 (*p* < 0.05, 95% CI (0.07, 0.10)). The proportions of specific agreements and individual kappa statistics per treatment are given in table 4.

**Table 4.** The kappa statistics and proportions of specific agreements for probabilistic recommendations per treatment plan type, along with their 95% asymptotic confidence intervals.

	specific kappa	specific agreement
1. surgery	0.17, (0.15–0.19)	0.43, (0.41–0.45))
2. radiotherapy	0	0
3. chemotherapy	0	0
7. sequential chemo-radio	0.06, (0.02–0.10)	0.07, (0.03–0.11)
8. concurrent chemo-radio	0.11, (0.03–0.19)	0.11, (0.03, 0.19)
9. induction chemo and surgery	0.01, (0–0.02)	0.01, (0–0.03)
10. neo-adjuvant chemo and surgery	0.06, (0.02–0.06)	0.06, (0.04–0.08)
11. surgery and adjuvant chemo	0.06, (0.04–0.08)	0.25, (0.23–0.27)

A clearly visible pattern in figure 9 is that the top treatment recommendations by the LCA BN almost exclusively comprise surgical treatment plans. If we focus on the non-surgical treatment plan columns, we see that the single modality plans: radiotherapy and chemotherapy are never recommended by the system, and the multimodal chemo-radiotherapy plans are recommended very rarely.

Furthermore, the ‘surgery’ row in figure 9 reveals that for the majority of the cases, the probabilistic decision support favours multimodality surgical treatment plans: 9, 10 and 11 over ‘surgery’ alone. Upon further analysis, we found that the 681 concordant cases were all early stage (Stage IA–IIB) patients, for whom surgery alone yielded marginally better survival expectancies compared with the multimodal surgical plans.

Finally, contrary to guideline rule-based decision support, the ‘induction chemotherapy before surgery’ and ‘neo-adjuvant chemotherapy before surgery’ treatment plans are recommended to a relatively high number of patients on the basis of maximizing the probability of 1-year survival. Nevertheless, it is evident that the maximum *a posteriori* estimations of ‘argmax_(T)_[*P*(Survival = Alive|Evidence, Treatment)]’ produce recommendations that are heavily biased towards surgical treatment plans and therefore do not concord with the recorded clinical practice.

### 3.2. Analyses with respect to TNM staging

**Figure 10.**
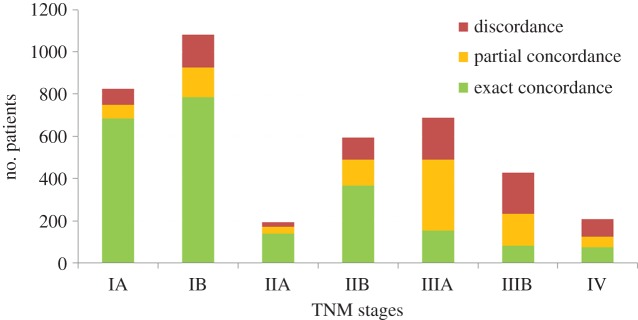
The exact and partial concordances between the guideline rule-based recommendations and the recorded treatment plans stratified with respect to TNM stages. (Online version in colour.)

In addition to our analyses of concordance based on treatment plan types, we also investigated the levels of exact and partial concordances with respect to the TNM stages of the test patients. Figure 10 shows the concordances between the guideline rule-based recommendations and the silver standards, stratified with respect to the TNM stages. It can be observed that the concordance rates are relatively high for early stage cancer patients. This may be explained by the limited variation between the disease specifics of early stage cancer patients and their corresponding treatment decisions.

On the other hand, concordance rates for locally advanced stage patients (Stage IIIA and IIIB) are significantly lower. This is not surprising since stages IIIA and IIIB comprise the widest variation in disease specifics (T and N stage combinations) among all TNM stages and constitute the patient group with the highest degree of uncertainty. As a result, treatment decisions tend to vary more with patient-specific differences.

One way to interpret the low exact concordance rates for the locally advanced stage patients, shown in figure 10, is that despite the more comprehensive rule coverage for these patients, the national and international guideline rules are not sufficient on their own to attain high levels of agreement between LCA recommendations and clinical practice. However, it should also be kept in mind that the silver standards, against which we compare our system recommendations, do not necessarily represent best practice patterns. Therefore, the relatively low concordance rates need not necessarily indicate deficiencies of our rule base. These can alternatively be interpreted as complex cases, which deviate from best practice recommended in the national and international guideline documents.

**Figure 11.**
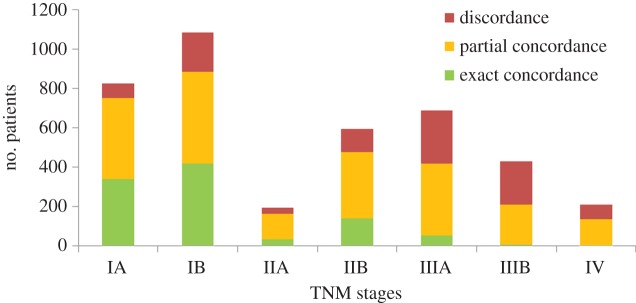
The exact and partial concordances between the probabilistic recommendations and the recorded treatment plans stratified with respect to the TNM stages. (Online version in colour.)

On the other hand, figure 11 reveals a different story for the probabilistic decision support results, with notably lower exact concordance results for the early stage patients. More strikingly, the exact concordance levels for locally advanced (IIIA and IIB) and advanced (IV) stage patients are close to zero. This may be attributable to the fact that while the LCA probabilistic recommendations favour surgical treatment plans regardless of the stage of the disease, in clinical practice the proportion of patients who are suitable for surgery decreases as the severity of the disease (judged by the TNM stage) increases.

It is clear from the results that the probabilistic decision support falls short in judging suitability for surgery, which should be determined by factors listed in §2.2.

## 4. Discussion

This paper presents the implementation and performance of a novel CDS prototype, LCA, which combines guideline rule-based and probabilistic decision support in order to assist the treatment selection decision of lung cancer experts in MDT meetings. Our results highlight the relative strengths and weaknesses of the guideline rule-based and probabilistic inference in providing decision support to the clinicians.

A direct comparison of the empirical concordance results achieved with guideline rule-based and probabilistic decision support reveals that the former performs better in simulating the recorded treatments in the database. While a high concordance rate with the recorded treatments does not necessarily imply better decision support, the rule-based CDS results at least provide sufficient evidence that the system is capable of making sensible personalized recommendations.

On the other hand, the relatively poorer concordance results of the probabilistic recommendations can be explained by the inability to incorporate additional factors, other than maximizing survival expectancy, into our probabilistic queries. While we stress that this shortcoming is not methodological but is due to lack of data on such factors in LUCADA, it is obvious that the usefulness of the treatment recommendations provided on the basis of survival maximization is limited. However, the posterior distributions can still be very informative in allowing the clinicians to compare the direct impacts of different treatment plans on survival expectancies.

**Figure 12.**
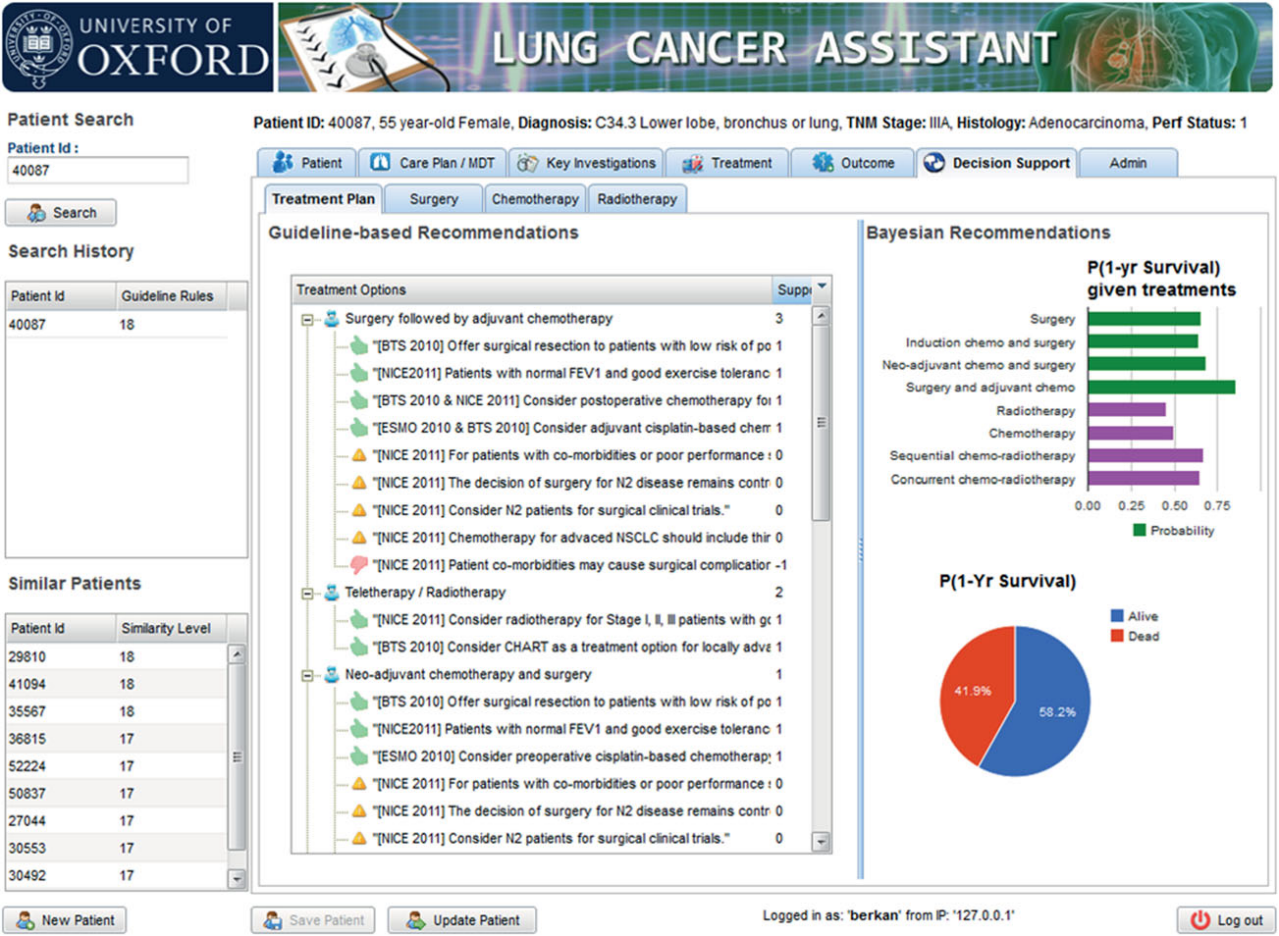
A screenshot of the Decision Support tab of LCA, where guideline rule-based (on the left) and probabilistic (on the right) decision support are provided together. (Online version in colour.)

The LCA user interface (UI) is designed to operate as an electronic patient form that includes all LUCADA data fields. Some MDTs, like the lung cancer MDT in the John Radcliffe Hospital in Oxford, already use electronic forms to record patient details prior to and during the meetings. LCA can potentially replace such electronic forms to provide instantaneous decision support upon entering a new patient or updating the details of an existing patient. The LCA UI displays guideline-based and probabilistic recommendations side by side as shown in figure 12. On the left, the supporting guideline-based arguments are symbolized with ‘thumbs up’ icons, whereas the opposing arguments are presented with ‘thumbs down’ icons. On the right, personalized 1-year survival expectancies and probabilistic treatment recommendations are displayed.

While we contend that the two decision support approaches are complementary, the relatively better concordance results achieved by the guideline rule-based CDS is due to their qualitative nature, which allows them to implicitly accommodate factors other than survival maximization in their recommendations. Therefore, in the absence of comprehensive electronic patient data, guideline rule-based CDS clearly serves the important purpose of laying out a more complete picture of the factors that govern treatment selection decisions.

However, more often than not the qualitative nature of guideline rules manifests itself in the form of vagueness and uncertainty in rule eligibility criteria. This can result in varied interpretations of guideline rules, raising the possibility of increasing practice variation despite apparent guideline adherence [46]. This major bottleneck in explicating the implicit expert knowledge can be addressed by the promotion of clear guideline authoring, keeping in mind the computerization steps and discouraging the use of vague terms [47]. Furthermore, the introduction of a ‘de facto standard’ and open source language, such as OWL2, for implementing guidelines would facilitate the dissemination and re-use of information between different CIG formalisms [48].

One of the major drawbacks in the wide adoption of guideline-based CDS is the need for manual elicitation and maintenance of rule-bases by clinicians and informaticians. In contrast to this dependency, the probability distribution underlying a BN is automatically updated as new patient data are added. This adaptive nature of the BN provides a more autonomous model that can evolve as more data are added.

A common treatment selection pattern observed in both guideline rule-based and probabilistic recommendations is that, compared to recorded decisions, they favour multimodality treatment plans over single modality ones. Similarly, both CDS recommendations over-prescribe surgical treatment plans compared to recorded clinical practice. Unless there are strong contraindications, such as metastatic disease, poor performance status or low lung capacity, the rule-base of LCA prioritizes single or multiple modality surgery plans for early and locally advanced cancer patients. The ‘over-prescription’ of surgery by the probabilistic decision support, on the other hand, is caused by the limitations of the one-dimensional analysis based on survival maximization. In either case, evidence from NLCA and the Society of Cardiothoracic Surgeons show that although the curative resection rates in the UK are rising, they are still not at optimum levels [49,50]. In this respect, the adoption of LCA, which lays out qualitative and quantitative indicators in support of surgical treatment plans at the MDT meetings, may help in optimizing resection decisions.

Nevertheless, we recognize that the research presented here is not without limitations. First, we recognize that the treatment plan selection, by nature, is a multi-faceted decision problem, involving complex criteria other than survival maximization, such as post-treatment quality of life assessment, access to treatment equipment and staff, and cost of treatment, among many others. From a patient-centred perspective, the MDT decisions also need to reflect the patient's views, preferences and circumstances [51]. Data on some of these concepts are very hard to capture, let alone quantify and put in a computer model. In an attempt to assist the expertise and holistic judgement of the clinicians, LCA only focuses on a subset of the more easily quantifiable biomedical aspects. As more diverse data become available, well-established decision analytic methods—not least decision networks [52]—can be used to calculate the expected utilities of decision alternatives using multi-criteria decision models.

Second, owing to the lack of data on 5-year survival rates, we adopt 1-year survival as our outcome measure. This is an informed decision, supported by literary evidence [15,16] and our analyses are of relevance to most patients (67%) who were dead within 1 year of diagnosis. However, it is possible that the probabilistic recommendations may change if a 5-year survival cut-off can be used. This will become possible with the continuation of LUCADA data collection.

Third, our experiments are based on retrospective data and as such may reflect biased treatment patterns. In order to validate the results prospectively, a properly conducted pilot study, which would span a minimum of 5-years and involve randomized control groups, would be necessary.

Finally, the integration of probabilistic and rule-based inference within LCA is only at the UI level. An obvious avenue for further research is coupling the outputs of the BN with the guideline inference engine. Williams & Williamson [53] have proposed such a proof-of-concept system that uses posterior probabilities obtained by Bayesian inference to weigh up competing arguments.

## 5. Conclusion

Practice variation and poor decisions in MDTs are inevitable, because clinicians have to make life or death decisions on phenomenally complex problems under very difficult conditions and with very limited support [54]. Computers can act as ever-attentive personal assistants to clinicians, and LCA is aimed to demonstrate how different decision support approaches, which derive from fundamentally different research hypotheses, can be used to complement each other to this end. We note that for LCA to be adopted in daily clinical practice, ensuring seamless integration with the workflow of the clinicians is of paramount importance. Once this is achieved, we believe it to have great potential in improving the quality of clinical decision-making, reducing the variation in treatment rates between MDTs and ultimately improving outcomes for patients.
